# Jaborandi and Its Active Principle Pilocarpine

**Published:** 1878-07

**Authors:** 


Jaborandi and its Active Principle Pilocarpine.—Jaboranpi has been used in this country and in Europe during the last four years to a considerable extent for its diaphoretic and sialogogue effects. But, remarkable enough while the preparations used have given entire satisfaction in the hands of many, severe complaints about the uncertainty of their action can be heard from others.

Can the difference in the action of—in my opinion—so valuable a drug be satisfactorily explained?

In Brazil where jaborandi has been used as a diaphoretic and—according to trustworthy reports—as a diuretic long before we knew anything about it, the leaves of the following plants are known as "Jaborandi:" piper jaborandi (Willdenon) Serronia jaborandi (Guillenica) Monniera trifolia (Aublet) and Pilocarpus pinnatus.

Who would expect to find that these different plants, which do not even belong all to the same species, possess exactly the same properties?

I have used the leaves of Pilocarpus pinnatus, the fluid extract made of them, and last, but not least, the active principle Pilocarpine in its combination with hydrochloric acid, known as hydrochlorate, or muriate of pilocarpine. I have found the action of jaborandi and its preparations in a great majority of cases reliable, quick and decided, and I do not hesitate to say that I consider jaborandi—derived from pilocarpus pinnatus—as the most powerful diaphoretic and sialogogue known at the present time.

The unpleasant disturbances, such as vomiting, headache, dizziness, fainting, colic, etc., are occasionally found to be produced by it, might be expected from such an active remedy, but, aside from slight gastric disturbances, I have never found any unpleasant symptoms to occur.

If I employ the leaves, I do it in the form of an infusion, which is prepared in the following way: Half an ounce of the leaves is coarsely powdered, and ten or twelve ounces of boiling water is poured over it. The infusion is kept at a temperature somewhat below the boiling point for about 15 minutes and strained. Of this I administer a small teacupful (warm) every two hours, and in the majority of cases I obtained good diaphoresis about ten minutes after the administration of the first dose. Of the different preparations of jaborandi which I have employed I obtained, generally speaking, the least satisfaction with the infusion. Nausea, vomiting, and sometimes headache, accompanied or followed in some cases the diaphoresis produced by the administration of the infusion of jaborandi.

The fluid extract which has probably been most frequently employed, has given entire satisfaction in all cases of adults. I employ the following simple formula:

R.—Ext. jaborandi fl (pilocarpus pinnatus)
Syr. simplex, aa ℥ ss.
MS. Teaspoonful at 2, 4, 6 and 8 P. M., and 8 and 10. A. M., 12 and 2 P. M. the next day.

The patient is to be kept in bed, well covered, and from 8 to 10 minutes after the administration of the first dose, its diaphoretic action takes place. After the second, and third dose, the diaphoresis is at its height. The flow of saliva commences according to my observations always after the diaphoretic action has begun, although it has been stated by others that the sialogogue influence of the drug becomes apparent before its diaphoretic action can be observed.

I advise the patient to be careful not to swollow the saliva, but to let it run from the mouth, and found with this little precaution that vomiting does not generally occur; if at all, it takes place with the 4th and 6th or 7th dose. Other disturbances I have not observed.

Hydrychlorate of pilocarpine, derived from the alkaloid found by E. Hardy in the leaves and in the root of pilocarpus pinnatus, is in many respects the most valuable of the preparations of jaborandi. It comes in small white crystals, very soluble in water, and is for different reasons especially adapted for hypodermic medication. Its action resembles that of the drug itself, but is more uniform and reliable than either the infusion or the fluid extract. It also influences the bronchial secretions by making them more fluid, and it has been used with advantage in croup, bronchitis, etc. A solution is made by dissolving ½ grain of hydrochlorate of pilocarpine in 30 minims of pure water. I use in cases of children from 6 to 10 years of age, 10 mins. of this solution, 1 1-6 gr. hypodermically and repeat the injection once or twice the next or following day. To adults I have given 20 mins. (⅓ gr.) repeated every day for three days.

The simplicity and almost painless manner of its administration, the fact that its hypodermical use does not cause any irritation, or abscess at the point of injection, the easy manner by which we are able to administer it in a state of uræmia, unconsciousness, during convulsions, etc., make it a most valuable remedy in the treatment of children. I used it in five cases of parenchymatus nephritis following scarlet fever, four of which occurred in children under 12 years of age, and I can only state that its action was very satisfactory, although it produced considerable vomiting in one and moderate emesis in another case.

Jaborandi deserves further trial and will be found of good service, when properly used, in cases of parenchymatus nephritis, general anasarca, pleurisy, bronchistis, etc. Prof. Marme Gottingen, states: atropine, in small doses, arrests all action of jaborandi, while large doses of pilocarpine cannot overcome active doses of atropine.—*Hospital Gazette.*

